# Biotic interactions outweigh abiotic factors as drivers of bark microbial communities in Central European forests

**DOI:** 10.1093/ismeco/ycae012

**Published:** 2024-01-25

**Authors:** Lukas Dreyling, Caterina Penone, Noëlle Valérie Schenk, Imke Schmitt, Francesco Dal Grande

**Affiliations:** Senckenberg Biodiversity and Climate Research Centre (SBiK-F), Frankfurt am Main 60325, Germany; Goethe University Frankfurt, Institute of Ecology, Evolution and Diversity, Frankfurt am Main 60438, Germany; Institute of Plant Sciences, University of Bern, Bern 3013, Switzerland; Institute of Plant Sciences, University of Bern, Bern 3013, Switzerland; Senckenberg Biodiversity and Climate Research Centre (SBiK-F), Frankfurt am Main 60325, Germany; Goethe University Frankfurt, Institute of Ecology, Evolution and Diversity, Frankfurt am Main 60438, Germany; Senckenberg Biodiversity and Climate Research Centre (SBiK-F), Frankfurt am Main 60325, Germany; Department of Biology, University of Padova, Padua 35122, Italy; National Biodiversity Future Center (NBFC), Palermo 90133, Italy

**Keywords:** microbial ecology, microbiome, forest, algae, bacteria, fungi, bark surface, community ecology, metabarcoding, environmental DNA

## Abstract

Bark surfaces are extensive areas within forest ecosystems, which provide an ideal habitat for microbial communities, through their longevity and seasonal stability. Here we provide a comprehensive account of the bark surface microbiome of living trees in Central European forests, and identify drivers of diversity and community composition. We examine algal, fungal, and bacterial communities and their interactions using metabarcoding on samples from over 750 trees collected in the Biodiversity Exploratories in northern, central, and southern Germany. We show that mutual biotic influence is more important than the abiotic environment with regard to community composition, whereas abiotic conditions and geography are more important for alpha diversity. Important abiotic factors are the relative humidity and light availability, which decrease the algal and bacterial alpha diversity but strongly increase fungal alpha diversity. In addition, temperature is important in shaping the microbial community, with higher temperature leading to homogeneous communities of dominant fungi, but high turnover in bacterial communities. Changes in the community dissimilarity of one organismal group occur in close relation to changes in the other two, suggesting that there are close interactions between the three major groups of the bark surface microbial communities, which may be linked to beneficial exchange. To understand the functioning of the forest microbiome as a whole, we need to further investigate the functionality of interactions within the bark surface microbiome and combine these results with findings from other forest habitats such as soil or canopy.

## Introduction

Forest ecosystems harbour a great diversity of microbial life in a variety of forest compartments, such as soil, dead wood, leaf surfaces, or bark surfaces [1]. Communities of microorganisms perform important functions in forests, including nutrient cycling and fixation, and symbiotic relationships with plants [2-4]. It is therefore crucial to understand the causes (and consequences) of microbial diversity changes in forests, i.e. understand their biotic and abiotic drivers. Ideally, we would know the complete microbial spectrum in all forest compartments, a task not nearly accomplished yet [1]. Here we contribute to closing this knowledge gap by focusing on a large but neglected forest compartment, the bark surface of living trees, and assessing the diversities, as well as biotic and abiotic drivers of bacterial, fungal, and algal bark surface communities.

Bark surfaces constitute one of the largest forest compartments [1]. They offer a multitude of micro niches for microbial colonization [5] and sustain diverse bacterial, fungal and algal communities [6-10] despite challenging environmental conditions such as low nutrient and water availability [6, 7, 11]. Microbial communities on bark are at the base of the forest food web, supporting animals, such as molluscs, mites, and lice [12-15] and macro-epiphytes such as mosses and lichens [16, 17]. According to Aschenbrenner et al. (2017) [16], these communities could also represent reservoirs of microbial taxa potentially “feeding” other forest compartments, e.g. via transmission by stemflow from the phyllosphere to soil [18].

Natural microbial communities engage in a wide variety of important interactions, ranging from the provision of nutrients [19] to parasitism [20]. Algae are primary producers, supplying these communities with photosynthetic products (e.g. [21]), but lack the ability to fix nitrogen, which, in turn, is often provided by mutualistic bacteria [22]. Other bacteria have been shown to be harmful to algae, e.g. by producing cell death inducing compounds [23, 24]. Fungi, on the other hand, are known to protect algae, for example from such harmful bacteria [24] but also from environmental stressors such as UV radiation [25]. They often provide a structural component for colonization through their filamentous nature, e.g. in lichens [26] or mycorrhiza [19]. In addition, the fungal mycelium has been proposed as a “transport path” for bacterial dispersal [27]. However, fungi and bacteria also engage in competition for nutrients [19]. Considering these types of interactions, it is highly likely that algae, fungi, and bacteria occurring in close spatial association on tree bark jointly influence each other’s diversity and community composition (e.g. [28]).

Abiotic conditions, which affect aboveground microbial communities of trees, have mostly been analysed with a focus on phyllosphere communities. For example, Liu et al. (2023) [29] found that lower biomass and species richness of phyllosphere algae in tropical forests is likely related to lower moisture retention on the leaf surface. Similar patterns can be observed for fungi and bacteria, e.g. in the phyllosphere of grapes, where the richness of fungi and bacteria increased with higher temperatures and rainfall [30]. Another important abiotic factor is exposure to UV radiation in aboveground habitats, which has been shown to alter bacterial community composition, but not population size, in the phyllosphere [31]. Based on these studies, we hypothesize that the bark surface microbiome, similarly to the phyllosphere, is affected by fluctuations in temperature, water availability, and UV radiation. For example, a previous study on bark microalgae from Mediterranean forests showed a higher abundance and diversity compared to those of temperate forests, likely due to differences in temperature and precipitation [32]. However, additional factors may contribute to shaping the unique communities on bark surfaces [7], for example, the age of the host tree [10, 33].

Interestingly, studies from soil microbial communities revealed that organismal groups occurring in the same habitat tend to exhibit different and sometimes opposite responses to abiotic changes. De Vries et al. [34] showed that bacterial networks are destabilized under drought conditions, while the effect on fungi was negligible. Even within organismal groups, responses can differ, as has been shown for drought responses of free living and mycorrhizal fungi [35]. To understand how abiotic conditions affect the microbiome, we thus need to include the full microbial spectrum captured at the same scales and time points.

Only few studies about natural bark micro-communities exist (e.g. [7, 8, 35]), and although they often included only single organismal groups [16, 36, 37], we already know parts of the diversity in bark surface communities [10]. However, to gain insights into how the whole community of microorganisms responds to present environmental change, and to model future changes, we need a comprehensive overview of the drivers behind community structure [1, 10]. Since diversity is multifaceted we need to go beyond alpha diversity and consider multiple diversity dimensions [38] to fully understand which biotic and abiotic factors shape community responses. Additionally, rare and common species might respond differently to the same drivers [39] and beta diversity can reveal homogenisation patterns [40].

In this study, we aim to elucidate how the environment structures multi-kingdom micro-organismal communities in one of the largest above-ground habitats of the terrestrial realm, the bark surface of forest trees. We sampled micro-communities from the bark of living trees, in 133 plots (over 750 trees) along a south-west to north-east gradient across Germany and assessed the relative contribution of abiotic (e.g. climate and forest features) and biotic (i.e. co-occurrences) factors in predicting the metabarcoding-derived diversity of three major microbial domains, i.e. terrestrial green algae, fungi, and bacteria. Specifically, we aimed at answering the following questions:

1) What is the alpha diversity and community composition associated with bark surfaces in Central European temperate forests?

2) What are the drivers of alpha diversity and composition of the bark surface microbial community? And specifically, what is the relative importance of biotic and abiotic factors?

## Material and methods

### Study design

We collected samples in May 2021 from the full set of 150 forest plots established by the Biodiversity Exploratories in three regions across Germany [41]. We defined a 20 m × 20 m subplot of the original 100 × 100 m^2^ plot and collected a composite sample of bark surface swabs from six trees per plot. Prior to sampling, we determined the most abundant tree species for each plot, based on a forest inventory [42]. All six trees sampled in each plot belonged to the predominant species. Some plots were excluded prior to the analysis, either because the dominant tree species did not occur in enough plots necessary for robust statistics, the plot was clear cut before sampling or because the extraction did not yield enough DNA. The dataset used for analysis contained 133 plots of the original 150 sampling plots. The dominant tree species in the final subset of 133 plots were beech (*Fagus sylvatica*), pine (*Pinus sylvestris*), or spruce (*Picea abies*). Following a previous study [10], the composite sample included two small (5–15 cm diameter at 150 cm height), two medium (15–30 cm), and two large (> 30 cm) trees. When no equal sampling was possible, we chose the size class that best represented the surrounding forest (36 of 133 plots, ~27%).

The sampling technique is described in detail in [10] with the only difference that all swabs from one plot were pooled together. In brief: we sampled the microbial community of the bark surface by swabbing with nylon-flocked swabs (FLOQSwabs™, Copan, Brescia, Italy) once around the stem at 150-cm height. The swabs were fixed in nucleic acid preservation (NAP) buffer [43] and stored at 4°C until DNA extraction. We included three extraction blanks (one per region) of six swabs exposed to ambient air. These were processed as if they were a biological sample.

### DNA extraction

As described in [44], samples stabilized in DNA preservation buffers need extra processing before extraction. To allow liberation of all material, including bacterial cells, we added an equal amount of phosphate buffered saline to the tube containing the swabs in NAP buffer. Afterwards, we moved the contents to a 50 ml tube (to allow movement) and vortexed the swabs for 30 s to dislodge material. We transferred 1.5 ml of the suspension to a 2 ml tube, centrifuged it at 6000× g for 15 min. and discarded the supernatant. We used an extraction kit (Quick-DNA Fecal/Soil Microbe Microprep, Zymo Research Europe GmbH, Freiburg, Germany). Modifying the protocol, we added the beads and buffer directly to the centrifugation pellet. Samples were shaken for a total of 6 min (SpeedMill PLUS, Analytik Jena, Jena, Germany). All subsequent steps followed the manufacturer’s protocol. DNA extracts were stored at −20°C.

### PCR amplification and high-throughput sequencing

We amplified algal, fungal, and bacterial DNA with universal primer pairs, targeting the ITS2 (ITS-Cha3 (CAACTCTCRRCAACGGATA) [45] and ITSu4 (RGTTTCTTTTCCTCCGCTTA) [45] for algae; FITS7 (GTGARTCATCGAATCTTTG) [46] and ITS 4 (TCCTCCGCTTATTGATATGC) [47] for fungi) and 16S V3-V4 (341F (CCTACGGGWGGCWGCAG) [48, 49] and 785R (GACTACHVGGGTATCTAATCC) [50] for bacteria) regions. We used double-index multiplexing with both primers being tagged by an octamer, allowing us to amplify all samples in triplicate. Each technical replicate included eight PCR negative controls as well as 23 empty wells as so-called “multiplex controls” to detect potential primer jump [51]. PCR reactions with a volume of 15 μl contained 5 ng of DNA, 7.5 μl of MyTaqTM HS Mix, 2x (Bioline GmbH, Luckenwalde, Germany), 0.6 μl 10 mM of each primer, and 4.3 μl DNAse free water. All samples were randomly placed on two 96-well plates, sharing the placement scheme between replicates. The cycling conditions differed between the organismal groups (Table 1).

**Table 1:** Cycling conditions for all three organismal groups. Differences are highlighted in bold. A = algae, F = fungi, B = bacteria.

**Phase**	**Temperature (**°C**)**	**Duration (s)**	**Number of cycles**
Initial denaturation	95	60	1
Denaturation	95	15	**A: 30** **F: 35** **B: 30**
Annealing	**A: 54, F: 56, B: 59**	15	
Elongation	72	10	
Final extension	72	60	1

After PCR, we cleaned each sample with magnetic beads (MagSI-NGSPREP Plus, magtivio B.V., Geelen, Netherlands) and measured DNA concentration using the Qubit dsDNA HS assay with a Qubit 3.0 (Thermo Fisher Scientific, MA, USA). Triplicates were pooled equimolarly and sent to Fasteris SA (Plan-les-Ouates, Switzerland) for library preparation (MetaFast protocol) and sequencing. Amplicons were sequenced with 2 × 300 bp paired-end reads on an Illumina MiSeq machine (Illumina Inc, San Diego, CA, USA).

### Bioinformatics

We trimmed the primers and demultiplexed the reads using Cutadapt (version 3.3., [52]). We used DADA2 (version 1.12.1, [53]) for filtering and trimming, denoising and sample inference to obtain amplicon sequencing variants (ASVs). For fungi and bacteria, we used DADA2 *assignTaxonomy()* and the publicly available databases UNITE general fasta release 9.0 [54], including eukaryotic ITS as outgroups, and SILVA 138.1 SSU Ref NR 99 [55]. For algae, no such database exists and we used the NCBI nt database (generated on 25 April 2022) with a local call to BLASTn. Afterwards we used the *taxonomizr* R package (version 0.8.0, [56]) to assign taxonomy. BLAST hits from uncultured or environmental origin, and below 95% identity were excluded. Reads were checked for contaminant sequences using *decontam* (version 1.16.0, [57]). The resulting ASV table was curated with the LULU algorithm (version 0.1.0, [58]), which is a tool for post-clustering curation based on co-occurrence of similar sequences and merges potential parent and child sequences. A table tracking the raw number of ASVs through the curation can be found in Supplementary Table 1.

### Analyses

We used R (version 4.2.2, [59]) together with RStudio (version 2022.12.0.353, [60]) to perform all the analyses. Data were combined with *phyloseq* (version 1.40.0, [61]). All graphics were generated with *ggplot2* (version 3.4.0, [62]) and *ggpubr* (version 0.5.0, [63]). To visualize community composition, we created relative abundance barplots of the 25 most abundant orders with the *microbiome* [64], *fantaxtic* [65], and *microViz* [66] packages. To avoid a loss of data, samples were not rarefied [67]. All analysis scripts are available at Zenodo [68] under doi: 10.5281/zenodo.10200121.

### Diversity

In order to capture multiple dimensions of diversity, we calculated Hill numbers [69, 70] (or effective species (in this case, ASV) number) for both alpha and beta diversity using the *hillR* package (version 0.5.1, [71]). In general, the weight given to the abundance (counts) of a taxon increases with Hill number. According to the definition by Chao et al. (2014) [70], Hill numbers measure the diversity of “all” (*q* = 0), of “typical” (*q* = 1) and “dominant” (*q* = 2) species (ASVs). In the following sections we use these terms when referring to the according q values.

We chose the first three levels of *q* = 0, 1, and 2 to have a direct comparison to widely used indices. For alpha diversity these correspond to species (ASV) richness (*q* = 0), Shannon entropy (*q* = 1), and inverse Simpson index (*q* = 2). Since beta diversity is inherently a comparison between two spatially separate populations [72] we calculated Sørensen-type similarity between pairwise communities as the CqN measure [73, 74]. To mirror the metrics used for alpha diversity, we calculated measures of Sørensen dissimilarity (*q* = 0), dissimilarity of the Horn index (*q* = 1), and dissimilarity of the Morisita–Horn index (*q* = 2).

### Environmental influence on diversity

To assess the effect of the environment on the microbial bark communities we chose a set of explanatory variables, based on prior hypotheses of how they might influence the community. Table 2 gives an overview and explains what the variables represent and how they were measured. All variables were scaled to standardize effect sizes and make them comparable. An overview of estimates can be found in Supplementary Table 2. We tested for significant differences in the tree-dependent variables between the tree species using a multivariate analysis of variance. Based on the results (Pillais trace = 0.86, *F* = 19.33, *P* < 0.001) and biological interpretation, we hypothesize that the host tree species (where the sample was collected) and tree-dependent variables (see Table 2) represent the same processes. Thus, we excluded the host tree species from the analysis. A table giving an overview of the environmental conditions can be found in Supplementary Table 3.

**Table 2:** Drivers of community composition tested in this study. Given are the tested variables, their measurements, descriptions, and data sources, as well as the direction of the effects obtained from linear models of β-diversity. Only the direction of significant (*P* < 0.2) effects from the Generalized Dissimilarity Models are shown. A = algae, F = fungi, and B = bacteria. *q* = 0, 1, 2 correspond to the Hill number. All datasets can be found in the “Biodiversity Exploratories Information System (BExIS)” at https://www.bexis.uni-jena.de/.

**Variable**	**Measurement**	**Proxy for**	**Source**	**Effect direction (β-diversity)**								
				***q* = 0**			***q* = 1**			***q* = 2**		
				**A**	**F**	**B**	**A**	**F**	**B**	**A**	**F**	**B**
**Abiotic factors:**												
Relative humidity	Relative humidity at 2 m above ground measured using a Rotronic HC-S3 probe. Averaged over the 2 weeks before and the week of sampling.	Water availability from the air.	BExIS dataset ID 19007 accessible through the public climate data search	↑				↑			↑	
Temperature	Air temperature in °C at 2 m above ground measured using a Rotronic HC-S3 probe. Averaged over the two weeks before and the week of sampling.	Growing conditions.	BExIS dataset ID 19007 accessible through the public climate data search	↓	↑	↑		↑	↑		↑	↑
Average DBH	Average diameter at breast height of all trees in the plot. Tree-dependent variable.	Average age of the trees.	BExIS dataset ID 22766									
Canopy openness	Percentage of pixels classified as “Sky” after LIDAR scanning. Tree-dependent variable.	Light availability.	BExIS dataset ID 27828		↑	↑		↑	↑		↑	↑
Gini coefficient	Mean heterogeneity of the tree size in the plot. Tree-dependent variable.	Mixture of old and young trees.	BExIS dataset ID 22766			↑			↑			↑
Stand density	Based on basal area (m^2^/ha). Measures the area of the plot is covered by tree trunks. Tree-dependent variable.	Connectedness of the potential habitat.	BExIS dataset ID 22766						↑			↑
Ratio of dominant trees	Percentage of the most abundant tree species in the plot. Tree-dependent variable.	Availability of suitable host trees for sampled communities.	Based on BExIS dataset ID 21426						↓			
Forest area	Percentage of forest in a 2000 m^2^ buffer around the plot.	Connection at the landscape scale.	BExIS dataset ID 15929								↑	
**Geographic factors**												
Region (LM) and geographic distance (GDM)	Distance between the plots.	Regional differences in available recruitment pool.	BExIS dataset ID 1000									
**Biotic factors**												
Algal diversity	Measured as Hill number *q* = 0, 1, and 2.	Biotic interactions.	Calculated from BExIS dataset ID 31511		↑	↑		↑	↑		↑	↑
Fungal diversity	Measured as Hill number *q* = 0, 1, and 2.	Biotic interactions.	Calculated from BExIS dataset ID 31510	↑		↑	↑		↑	↑		↑
Bacterial diversity	Measured as Hill number *q* = 0, 1, and 2.	Biotic interactions.	Calculated from BExIS dataset ID 31512	↑	↑		↑	↑		↑	↑	

We used multiple linear regression models to study the responses of alpha diversity to abiotic and biotic factors. All models were specified as follows:

*lm(biotic Y ~ region + relative humidity + temperature + average DBH + canopy openness*

*+ gini coefficient + stand density + ratio of dominant trees*

*+ forest area + biotic1 + biotic2 + offset(library size)).*

Where biotic 1 and 2 represent the alpha diversities of the two other groups (e.g. when bacteria are the response variable, fungi and algae are biotic 1 and 2). Biotic influences were always modelled on the same diversity level, e.g. the response of algal q0 to changes in bacterial and fungal q0. The linear models included an offset term to account for the variation in library size between the samples.

To correct for multiple testing, we corrected the *P*-values for type 1 errors with a Benjamini–Hochberg correction and a threshold of *P* < 0.2. Combining this relaxed threshold and correction allows us to detect effects on this unknown system we would otherwise miss while still being cautiously optimistic that the effect is not a false positive.

To model changes in beta diversity we used Generalized Dissimilarity Modelling (GDM, [75-78]) fit through the *gdm* package [79]. GDM models pairwise dissimilarities between plots taking non-linear relationships into account, e.g. rates of change can be more rapid at some points along a gradient. This allows us to observe patterns of non-linearity common in ecology [78]. GDM follows a similar structure as Generalized Linear Models, but instead of taking individual explanatory values and assessing their effect on the response variable (here the beta diversity) it models the absolute difference between a pair of values, ordered along the explanatory variable’s gradient. For example: if Plot1 has an average temperature of 5°C and Plot2 of 9°C, then the GDM takes the difference of 4°C to (non-linearly) model how dissimilar the microbial communities of the two plots are, at the gradient between 5°C and 9°C. The explanatory variables mirrored the linear models of the alpha diversity analysis but included the geographic distance between plots instead of the region identity. The variation in library size between samples was incorporated into the GDM as a weighting factor, putting less weight on larger differences. *P*-values for GDMs are calculated based on a permutation procedure (*n* = 100 permutations) and were also corrected for multiple testing using the Benjamini–Hochberg correction with a threshold of *P* < 0.2.

## Results

### Diversity

We found a total of 131 ASVs for algae, 1750 for fungi, and 1263 for bacteria. The highest ASV richness at the regional level occurred within fungi, while the lowest number of ASVs was found for the terrestrial green algae (Table 3). Generally, we observed that ASV richness decreases from south-west (Swabian Alb) to north-east (Schorfheide-Chorin) Germany (Table 3).

**Table 3:** Number of ASVs per organism group in total, and split by study region.

**Organism group**	**Total # of ASVs**	**Swabian Alb (south-west)**	**Hainich-Dün (central)**	**Schorfheide-Chorin (north-east)**
**Algae**	131	99	100	76
**Fungi**	1750	1123	775	763
**Bacteria**	1263	787	666	541

**Figure 1:**
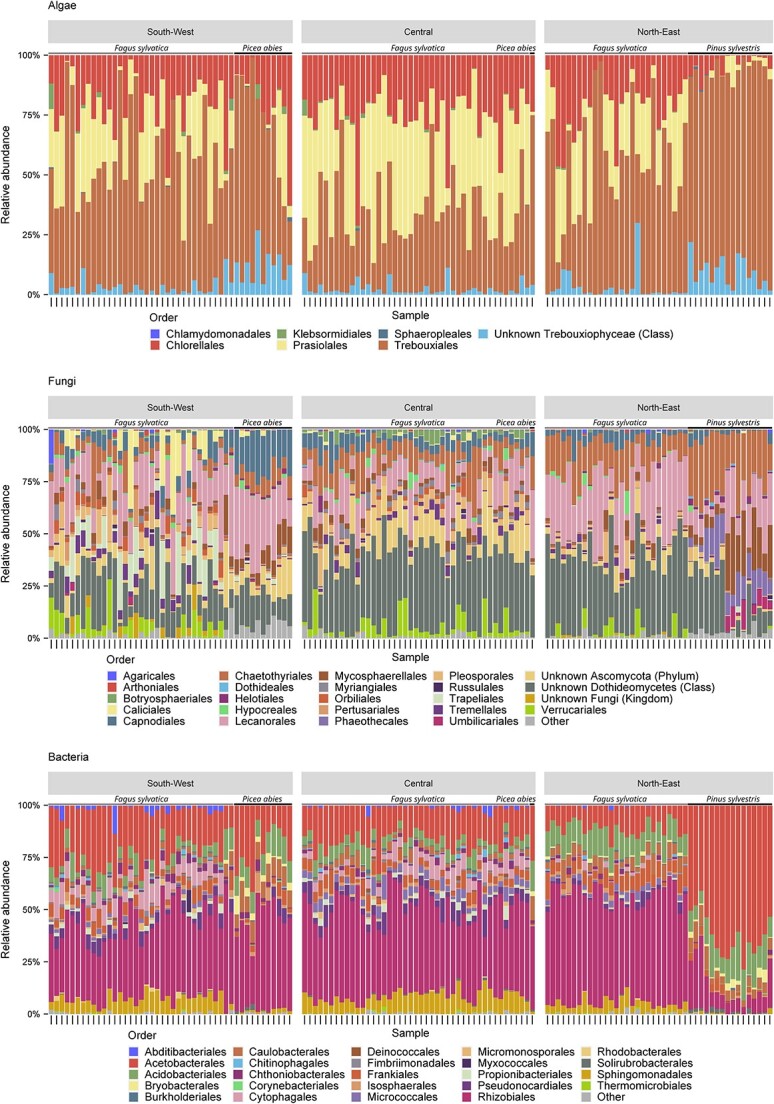
Community composition of algal, fungal, and bacterial communities associated with bark surfaces in three study regions. Given is the relative abundance of orders per plot. Each bar represents one plot. Only the 25 most abundant orders per organismal group are included.

The most abundant algal orders were Trebouxiales, Chlorellales, and Prasiolales (Fig. 1). The bacterial portion was primarily composed by taxa from the order Rhizobiales followed by Acetobacterales, which became dominant on some plots in the north-east (Fig. 1) where pine was the dominant tree species. For fungi, we found a different pattern, with a large proportion of reads not assignable at the order level, predominantly stemming from unassigned Dothideomycetes. There were no dominating orders, with Lecanorales, Capnodiales, and Chaetothyriales showing the highest relative abundance in the assignable portion of reads (especially in the north-east). Generally, abundance patterns were similar across regions and plots, with the exception of pine-dominated plots.

### Drivers of diversity

#### Alpha diversity

**Figure 2:**
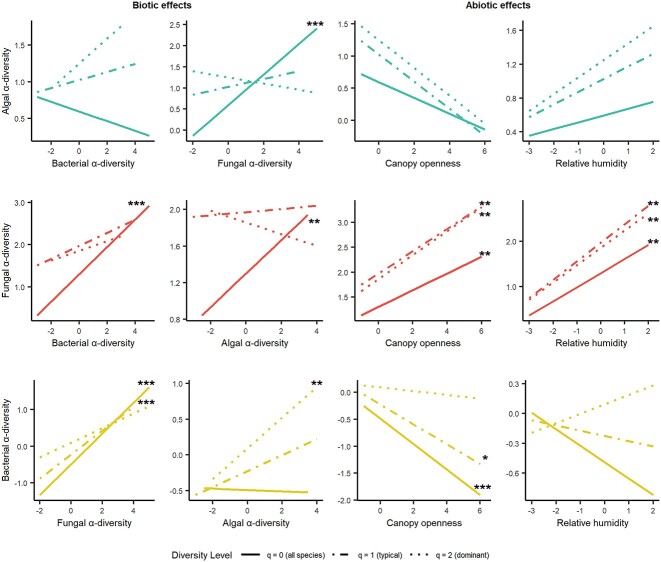
Drivers of algal, fungal, and bacterial communities associated with bark surfaces. Graphs indicate the effects of biotic and abiotic variables on three α-diversity measures (hill number *q* = 0, 1, and 2) of all three organismal groups. These are the results of linear models. Axes are scaled. Asterisks indicate Benjamini–Hochberg corrected *P*-values (^*^< 0.2, ^*^^*^<0.1, ^*^^*^^*^< 0.05). For an explanation of the variables, see Table 2.

All three organismal groups responded significantly (*P* < 0.2 after Benjamini–Hochberg correction) to biotic factors on at least one diversity level. Both algal and bacterial “all species” diversity (*q* = 0) significantly (both *P* < 0.01) increased with higher fungal ASV richness (Fig. 2), while there was no effect of algal ASV richness on bacteria and vice versa. Fungal ASV richness increased significantly with algal and bacterial ASV richness (algae *P* = 0.056, bacteria *P* = 0.001). Dominant algae (*q* = 2) positively influenced bacterial diversity (*P* = 0.078, Fig. 2). The mutual influence of dominant fungi and algae was negative, although not significant.

An important abiotic factor was canopy openness (proxy for light availability; Table 2), which influenced both fungal and bacterial diversity significantly. While fungal diversity increased with higher canopy openness (q0 *P* = 0.078, q1 *P* = 0.063, q2 *P* = 0.057), bacterial diversity of all and common species (ASVs) significantly decreased (q0 *P* = 0.005, q1 *P* = 0.143) (Fig. 2). A negative direction of algal diversity (*q* = 0–2) could be observed with increasing canopy openness. Relative humidity increased fungal diversity significantly, while decreasing directions were found for the diversity of all and typical bacteria while dominant bacteria increased. All levels of diversity for algae increased with higher humidity. The effects of the variables not shown here can be found in Supplementary Figure 1.

**Figure 3:**
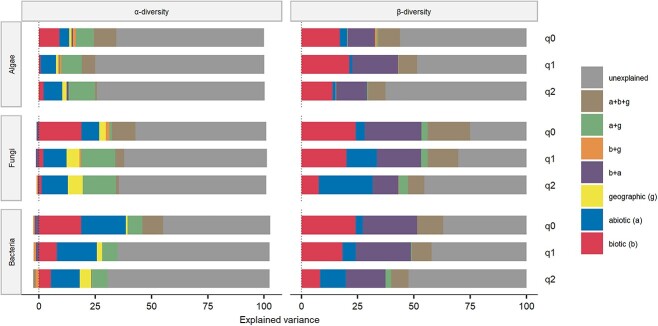
Contribution of different drivers to total observed variance in alpha and beta diversities. Abiotic variables (a), biotic variables (b), geographic distance (g), and combinations of these drivers explain part of the overall variance. Negative variance in the linear models is explained by the two factors having opposing effects on the response.

The variance (adjusted *R*^2^) explained by the linear models ranged between 17 and 48% (Fig. 3) and was the lowest for algal alpha diversity. The pure variance explained by abiotic factors was often higher than that of biotic factors on the alpha diversity level (Fig. 3), except when assessing diversity of “all” species. Abiotic factors also explained more variance when considered in combination with geographic factors (Fig. 3 a + g), especially for models of fungal and bacterial alpha diversity.

#### Beta diversity

**Figure 4:**
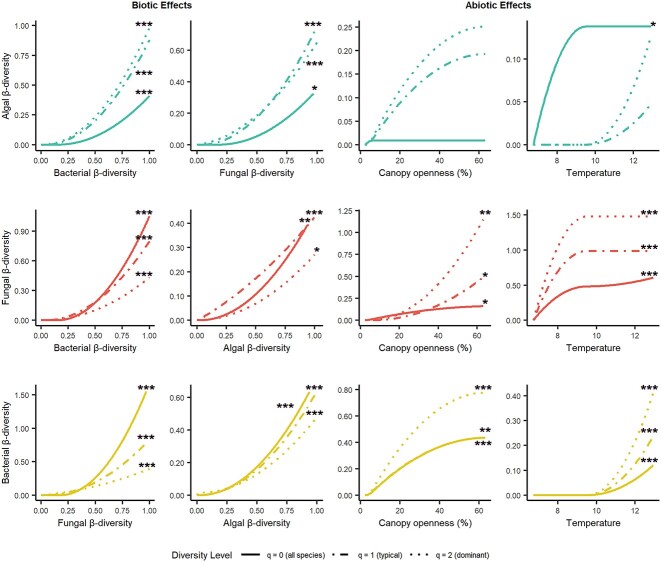
Drivers of algal, fungal, and bacterial communities associated with bark surfaces. Graphs indicate the effects of biotic and abiotic variables on three β-diversity measures (hill number *q* = 0, 1, and 2) of all three organismal groups. These are the results of GDM. The higher the curves maximum is, the larger the effect of the explanatory variable. The form and slope of the curve indicates how rapid changes in β-diversity are in relation to outside influences. Asterisks indicate Benjamini–Hochberg corrected *P*-values (^*^< 0.2, ^*^^*^<0.1, ^*^^*^^*^< 0.05). For an explanation of the variables, see Table 2.

The beta diversity of all three organismal groups responded significantly (algae all *P* < 0.05, except effect of dominant fungi *P* < 0.1; fungi all *P* < 0.05, except “all” and dominant algae *P* < 0.1; bacteria all *P* < 0.05) to changes in the diversity of the respective microbial partners (Fig. 4), e.g. if two plots differed in their bacterial community, they also differed in their algal and fungal composition. Most of the effect curves followed an exponential shape sloping upwards with increasing community dissimilarity, meaning the effects were strongest at high β-diversity and indicating a concurrent change of community composition. Assessing predictor importance (deviation in variance explained when a given predictor is permuted), bacteria were the most important biotic predictor on all three levels of fungal and algal beta diversity (Table 4), followed by fungi, which were slightly less important for algae and bacteria at all levels. Dominant bacteria were the most important biotic predictor for fungi, while algae were the most important biotic predictor of β-diversity of dominant bacteria.

**Table 4:** Importance of the tested variables for the prediction of algal (A), fungal (F), and bacterial (B) beta diversity on all three levels of *q*. The most important predictors are highlighted in bold; *asterisks* indicate significance of predictors after Benjamini–Hochberg correction in the GDM. ^*^P < 0.2, ^*^^*^P < 0.1, and ^*^^*^^*^P < 0.05.

**Variable**	**Predictor importance for β-diversity**								
	***Q* = 0**			***Q* = 1**			***Q* = 2**		
	**A**	**F**	**B**	**A**	**F**	**B**	**A**	**F**	**B**
**Biotic factors**									
Algal diversity	X	1.47^*^^*^	2.73^*^^*^^*^	X	3.42^*^^*^^*^	6.69^*^^*^^*^	X	2.79^*^	7.31^*^^*^^*^
Fungal diversity	3.30^*^^*^^*^	X	19.64^*^^*^^*^	8.67^*^^*^^*^	X	9.68^*^^*^^*^	10.28^*^^*^^*^	X	4.41^*^^*^^*^
Bacterial diversity	7.95^*^^*^	17.52^*^^*^^*^	X	**11.66** ^*^^*^^*^	10.32^*^^*^ ^*^	X	16.50^*^^*^^*^	5.20^*^^*^^*^	X
**Abiotic factors**									
Geographic	0.15^*^	0	0	0.04	0.01	0	0.36^*^^*^^*^	0	0
Relative humidity	2.57^*^	0.02	0	0	1.03^*^^*^	0.03	0	1.91^*^^*^	0.32
Temperature	1.89^*^	6.51^*^^*^^*^	0.85^*^^*^^*^	0.13	12.85^*^^*^^*^	4.31^*^^*^^*^	0.55^*^	27.15^*^^*^^*^	11.79^*^^*^^*^
Average DBH	0.23	0.06	0	0.23	0.03	0	0.55	0.03	0.09
Canopy openness	0.04	0.46^*^	2.51^*^^*^	**0.54**	1.33^*^	2.40^*^^*^^*^	**0.61**	4.31^*^^*^	6.82^*^^*^^*^
Gini coefficient	0.64	0.26	0.58^*^	0.41	0.25	1.02^*^^*^^*^	0.48	0.13	1.71^*^^*^^*^
Stand density	0	0.04	0.20	0.22	0.08	1.04^*^	0.38	0.10	3.15^*^^*^^*^
Ratio of dominant trees	0.78	0.07	0.13	0.28	0.03	0.51^*^	0.33	0.13	0.51
Forest area	0.66	0.19	0.05	0	0.33	0.36	0	1.40^*^	0.52

Of the abiotic factors we tested, temperature and canopy openness were the two most important predictors of community dissimilarity (Table 4), but their importance was usually lower than that of the biotic factors, except for typical and dominant fungi, as well as dominant bacteria. Differences in temperature significantly influenced the communities for most measures (Fig. 4, algae q0 = *P* < 0.2, bacteria and fungi all *P* < 0.05), but not the typical and dominant algal communities. For fungi, we found high community dissimilarity at lower temperature which saturated into homogeneous communities at higher temperatures (Fig. 4). Bacterial communities generally showed strong changes in dissimilarity at higher temperature, but beta diversity remained unchanged at low temperature (Fig. 4). Changes in canopy openness and the associated increase in light availability significantly influenced the communities of both fungi and bacteria at all levels of beta diversity (Fig. 4, fungi q0 + q1 = *P* < 0.2, q2 = *P* < 0.1; bacteria q0 = *P* < 0.1, q1 + q2 = *P* < 0.05). Bacterial communities displayed more rapid turnover with small increases in light availability in closed forests than in open ones (Fig. 4). The patterns of algal diversity were similar to those of bacteria and, although not significant, canopy openness usually was the most important abiotic predictor (Table 4; small effect size). Fungi responded differently to canopy openness. While the response of the full community was almost linear with shallow slope at increasing openness, the typical and dominant taxa showed a rapidly changing composition with high rates of change in more open conditions which is potentially connected to the strong increase in alpha diversity. The effects of the variables not shown here can be found in Supplementary Figure 2 and an overview of predictor importance can be found in Table 4.

The variance of beta diversity explained by the GDMs was between 37 and 75% and, again, was lowest in the models of algal diversity. In contrast to the alpha diversity, biotic factors explained more variance than abiotic factors in all three organismal groups, except for dominant fungi and bacteria (Fig. 3). A similar amount of variance was explained by combined effects of biotic and abiotic effects. Geographic distance explained only small amounts of variance.

## Discussion

### Fungi are the richest group found on bark surfaces with many unknown taxa

We studied the three main micro-organismal groups of the bark surface, and found that communities of fungi contained 90 ASVs per sample on average; 4.5 times more than algae (20 ASVs) and 1.3 times more than bacteria (70 ASVs). Compared to other forest habitats like soil, the microbial bark surface community is more unknown, especially for fungi. A study with soil samples from the same plots, sampled approximately at the same time, found that only 2% of the relative abundance came from fungal ASVs that were not assignable past the order rank [80], while in our study it was up to 50%, much of it from the ubiquitous class Dothideomycetes [81, 82]. The diversity harboured by bark surfaces, and especially its unknown portion, underlines the importance of further research on the bark microbiome. Of particular interest is the identification and potentially isolation of unknown fungi, considering that bark surfaces have been proposed as microbial reservoirs [16] that potentially contain an array of pathogenic and/or beneficial taxa relevant to plant health, as previously shown for bacterial epiphytes on grapevines [35]. Furthermore, it is likely that the reservoir effects of bark shape assembly processes in other forest compartments, e.g. by enabling early colonization of the phyllosphere in spring [8, 16] through the bark’s seasonal stability [83], or dispersal to soils via stemflow [18]. Additionally, it has been shown that bark is of great importance for the composition and diversity of deadwood microbiomes. In a study by Hagge et al. [84] bark coverage increased the importance of stochastic assembly mechanisms, one of which could be “priority effects” of the original community found in the bark surface reservoir.

### Abiotic conditions strongly impact alpha, but not beta diversity

To assess how the different groups within the bark surface microbiome respond to changes in their environment, we tested abiotic variables associated with climatic conditions and habitat connectedness. From previous studies of phyllosphere microbiomes, we expected that variables directly influencing individual organisms (e.g. humidity, temperature, light) would have the largest impacts on the communities. We found that the alpha diversity of the bark surface microbiome is strongly affected by abiotic factors, while abiotic factors (except temperature) were less important for community dissimilarity (Figs 3 and 4, Table 4). This indicates that while the size of the community may be limited by certain environmental conditions, which regulate ASV richness and how evenly common and rare taxa occur, they only weakly affect which taxa are present in the microbiome.

Since abiotic conditions are known to influence different components of micro-organismal communities differentially, e.g. bacterial soil communities responding more strongly to drought than fungal communities [34] and free-living fungi being more susceptible to drought than mycorrhizal fungi [85], we expected different responses of algae, bacteria, and fungi, especially to climatic conditions. When considering the three organismal groups, we found the strongest difference for humidity (alpha diversity) and temperature (beta diversity). While humidity strongly increased fungal alpha diversity, it had no significant effects in the other two organismal groups. However, bacterial diversity of “all” and “typical” species (ASVs) decreased with relative humidity. In contrast, temperature had no significant effect on alpha diversity at all. These contrasting effect directions for humidity are known from phyllosphere microbiomes [30], as well as from rhizosphere communities [86]. Strong differences even within organismal groups (e.g. [30, 85]) underline the need for caution when generalizing these patterns.

At the beta diversity level, temperature had the strongest effect on fungal communities and on dominant bacteria. Similar to the findings of de Vries et al. [34], we found that fungal communities were more similar at the high end of the temperature range, while the (typical and dominant) bacterial, as well as algal, communities showed higher turnover with increases in temperature. The exponential curve for algae and bacteria might indicate that these communities reach a “tipping point” after which rapid change sets in, accompanied by high turnover along the temperature gradient [78].

Light is expected to affect both photosynthetic organisms and microbial alpha and beta diversity [84, 87]. In the current study, a higher availability of light lead to an increase of fungal, but a decrease of algal and bacterial diversity (Fig. 2), suggesting that bark surface algae and bacteria are adapted to low light conditions and potentially damaged by too much UV radiation [31, 88]. For beta diversity, the slopes reached a plateau at higher light levels for both algae and bacteria, indicating homogeneous communities at higher light conditions. This suggests that the algal and bacterial communities become less rich but are potentially adapted to higher light conditions, for example, through the synthesis of carotenoid compounds [89]. Although many fungi employ strategies to limit damage from UV radiation, making them potentially more resistant to high light stress [90], we detected high rates of change under high light conditions for fungi. The high turnover, especially in the dominant fungal taxa, potentially indicates that fungal communities of open canopy forests are specialized towards high light availability.

### Biotic interactions determine community composition

Interactions between algae, bacteria, and fungi have been shown to take many forms, from competition over parasitism to well described symbioses like lichens [17, 19, 20, 24]. Thus, we expected close connections between the three organismal groups in the bark surface microbiome. Indeed, we could observe that changes in the community composition were highly depending on changes in composition of the other groups. This suggests that there are certain fractions of algal, fungal, and bacterial communities that are favourably associating with one another. Similarly, Arrigoni et al. (2018) [8] described a state of stable equilibrium between pathogenic and beneficial bacteria and fungi on the bark of fruit trees.

While biotic effects were less important for alpha diversity, they were in almost all cases far more important for beta diversity than abiotic effects and explained the most variance (Fig. 3). Fiore-Donno and colleagues [91] recently reported similar patterns within the alpine soil microbiome, where biotic interactions outweighed edaphic and topographic influences. Within the biotic factors, bacterial beta diversity was the most important. This might be due to the high pathogenic potential of bacteria affecting both algae and fungi [19, 24], but also due to supplying nutrients like nitrogen, especially considering the high impact on algal communities for which growth promotion through bacterial co-occurrence has been reported [22, 92]. Furthermore, heterotrophic bacteria commonly colonize the phyco- and mycosphere, the space surrounding algal and fungal cells [21, 93]. In these niches, they exchange compounds like photoassimilates and engage in other beneficial interactions with the host, mirroring the plant rhizosphere [21]. Bacteria, on the other hand, are strongly influenced by the fungal communities, potentially due to the fungal mycelium providing opportunities for transport [27] but also protective structures under non-favourable conditions [94], in addition to the provision of carbon from cell wall material [95]. Since close interactions between fungi and algae have been known for a long time, most notably from the lichen symbiosis [96], the strong fungal influence on algae was expected and was only slightly less than that of bacteria. A study by Hom and Murray [97] showed that mutualistic interactions of algae and fungi can also form spontaneously under low nutrient conditions, which are an inherent characteristic of the bark surface habitat.

It is important to note that a large proportion of the variance in the beta diversity models is jointly explained by abiotic and biotic conditions, and thus we cannot exclude indirect effects of abiotic conditions on a certain group through changes in another, e.g. increasing temperature could lead to changes in fungi which, in turn, changes bacterial and algal communities. However, we also need to consider that the microbiome can alter and mediate abiotic conditions such as nutrient or water availability [98, 99] as has been shown for microbiome–plant relationships (e.g. [100]).

### Regions differ mostly in their alpha but not beta diversity

Geographical distance is often associated with differences in environmental conditions. Previous studies of macro-organisms, like plants [101] and arthropods [102], in the Biodiversity Exploratories found significant differences between the three study regions that can be explained by nutrient availability, substrate differences, and land-use intensity. For the bark microbiome, we expected a similar pattern due to some differences in tree species (pine in the north-east, spruce in the south-west) and the corresponding differences in the direct abiotic environment. Similarly, we found significant differences for alpha diversity between the regions with a diversity decrease from the south-west to the north-east region. Region explained much of the variation in alpha diversity, but the high variation explained jointly with abiotic conditions suggests that there may be abiotic differences between regions not considered in our study, e.g. wind as a dispersal vector [103]. In previous studies of subaerial algae [32] and phyllosphere microbiomes [30] geographic location was a main influencer of microbial diversity leading to distinct communities. However, predictors were not as finely differentiated as in our study. We found that the geographic effect on community composition is negligible (Table 4), despite significant impacts on algae (Supplementary Table 2), mirroring the results of Aguirre-von-Wobeser et al. [9] for bacteria and fungi on avocado bark.

### Caveats

While our sample sites are representative of Central European forests [41] generalizations extending to other forest types, climate zones, or continents should be drawn with caution. Since the bark microbiome is still highly unknown at the global level we want to underline the need for further studies to make these comparisons possible. However, because similar forest features affect the diversity of multiple trophic groups in both temperate and tropical forests (e.g. [38, 104]), we hypothesize that this might also be the case for the bark microbiome. Additionally, we were not able to capture all possible forest parameters that might contribute to the community assembly, as evident from the percentage of unexplained variance. The tree species included in this study (*F. sylvatica, P. abies*, and *P. sylvestris*) vary in additional features like bark texture, pH or chemical composition, opening up other possibilities for niche differentiation even at the level of individual trees. Furthermore, there may also be a seasonal influence on the microbiome (e.g. [105]). Future studies would greatly benefit from including not only micro-niche parameters, but also spatio-temporal data, to clarify the driving mechanisms further.

Beyond the addition of further deterministic factors, the unexplained variance also warrants consideration of other, non-deterministic, assembly mechanisms at play. Zhou and Ning [106] state that deterministic and stochastic processes are both of great importance for microbial communities, and act in the same temporal space. Ecological stochasticity, including processes such as ecological drift, diversification, death, and “birth”, but also random colonization events, are certainly also influencing the bark microbiome. These processes are highly likely to shape the community composition and manipulate the connection of the bark surface microbiome with other forest compartments, e.g. through dispersal or “priority effects”. Indeed, bark has already been shown to increase the importance of stochastic processes for community dynamics in deadwood microbiomes, especially in the early colonization stage [84].

Another important aspect is of technical nature: the use of ASVs over Operational Taxonomic Units (OTUs). It is an ongoing debate which approach is more meaningful for obtaining diversity estimates, especially for fungi. No consensus has been reached to date, with recent studies making compelling statements for either choice [107-110]. We decided to use ASVs because of the higher accuracy without imposing arbitrary thresholds. To account for possible slight variations even within individuals and species, especially when considering ITS as a marker, we employed best-practice tools like the LULU algorithm [58]. Diversity estimates of any metabarcoding study, in our opinion, should be interpreted with care and taken as the diversity of sequence variants.

## Conclusions

In this study, we provide the first comprehensive assessment of the bark surface microbiome and its drivers in Central European forests. Our results can inform future hypothesis-driven research such as predictive modelling to assess the responses of the forest microbiome to future environmental conditions under climate change. We show that while abiotic factors influence the microbial communities, biotic interactions are usually more important, especially for community composition. Our study highlights the importance of integrating research on a diverse array of organisms if we want to understand the processes governing microbiome assembly. Combining our findings with results from other forest compartments will allow us to assess which taxa are shared between microbial habitats in forests and study how connections as well as dispersal, e.g. through stemflow, function among them. Lastly, future studies will benefit from the addition of functional information, e.g. through meta-transcriptomics or -genomics, since the nature of the interactions remains hidden and difficult to identify.

## Supplementary Material

Supplementary_figure1_effects_alpha_supplementary_ycae012

Supplementary_figure2_effects_beta_supplementary_ycae012

Supplementary_table1_ycae012

supplementary_table_2_ycae012

supplementary_table3_ycae012

## Data Availability

The raw reads have been deposited in the NCBI BioProject database (https://www.ncbi.nlm.nih.gov/bioproject/) under BioProject accession number PRJNA932736, SRA numbers SRR23371988 – SRR23371990. Species list, ASV tables, and metadata can be found in the Biodiversity Exploratories Information System (BExIS) (https://www.bexis.uni-jena.de/) under Dataset IDs 31506, 31508 – 31512. Additional datasets used in, but not generated for this study, are also available from BExIS under the accession numbers stated in Table 2. Dataset 19007 refers to the climate data that can be exported via the public climate data tool at https://www.bexis.uni-jena.de/). Furthermore, additional figures and tables supplementing the analysis can be found in the Supplemental Material. The full script as well as intermediate data needed to replicate the analysis are available at Zenodo [68] under doi: 10.5281/zenodo.10200121.
