# Supplementary material for: Biotic interactions outweigh abiotic factors as drivers of bark microbial communities in Central European forests
Source: ISME Commun. 2024 Jan 25;4(1):ycae012. doi: 10.1093/ismeco/ycae012 (PMC10945369; doi:10.1093/ismeco/ycae012)
Supplement: Supplementary_table1_ycae012 [file supplementary_table1_ycae012.pdf]

Supplementary Table 1: ASV numbers tracked through our assignment and data cleaning pipeline. Our sequencing runs contained DNA from soil and bark samples that thus needed to be jointly processed until they could be split after curation and filtering.

	Algae	Fungi	Bacteria
**ASV table processing**			
Raw ASVs	12,911	18,300	9,692
After contaminant removal	12,896	18,280	9,684
After LULU curation	8,091	11,981	4,352
**Taxonomy table processing**			
Before filtering	12,340	18,300	9,692
After filtering uncultured assignments	4,994	Not applicable	Not applicable
After filtering at 95% similarity	3,153	Not applicable	Not applicable
After filtering for organism group	966	16,385	7,131
**Combining the tables**			
After combining curated ASV table and taxonomy table	536	9,645	3,019
Taxa in bark samples (included in this study)	131	1,750	1,263
Taxa in soil samples (excluded from this study)	514	8,680	1,815
